# Supplementary material for: Distinct polymorphisms in a single herpesvirus gene are capable of enhancing virulence and mediating vaccinal resistance
Source: PLoS Pathog. 2020 Dec 11;16(12):e1009104. doi: 10.1371/journal.ppat.1009104 (PMC7758048; doi:10.1371/journal.ppat.1009104)
Supplement: S1 Table — (DOCX) [file ppat.1009104.s004.docx]

| ***meq* isolated** | **Genbank accession** | **Recombinant virus** | **Genbank accession** |
| --- | --- | --- | --- |
| CVI988/Rispens | AY243335.1 | vacMeq | MT797630 |
| JM/102W | HM488348.1 | vMeq | MT813453 |
| 617A* | AY362712.1 |  |  |
| RB-1B | AY243332.1 | vvMeq | MT797629 |
| N | AY362718.1 | vv+Meq | MT797631 |

* *meq* gene and recombinant virus used in the biological replicate (Fig. S1)
