# Supplementary material for: Distinct polymorphisms in a single herpesvirus gene are capable of enhancing virulence and mediating vaccinal resistance
Source: PLoS Pathog. 2020 Dec 11;16(12):e1009104. doi: 10.1371/journal.ppat.1009104 (PMC7758048; doi:10.1371/journal.ppat.1009104)
Supplement: S2 Table — (DOCX) [file ppat.1009104.s005.docx]

**CVI Meq (vacMeq)**

Consensus MSQEPEPGAMPYSPADDPSPLDLSLGSTSRRKKRKSHDIPNSPSKHPFPDGLSEEEKQKL 60

CVI ............................................................ 60

CVI seq 1 ............................................................ 60

CVI seq 2 ............................................................ 60

CVI seq 3 ............................................................ 60

Consensus ERRRKRNRDASRRRRREQTDYVDKLHEACEELQRANEHLRKEIRDLRTECTSLRVQLACH 120

CVI ............................................................ 120

CVI seq 1 ............................................................ 120

CVI seq 2 ............................................................ 120

CVI seq 3 ............................................................ 120

Consensus EPVCPMAVPLTVTLGLLTTPHDPVPEPPICTPPPPSPDEPNAPHCSGSQPPICTPPPPDT 180

CVI ............................................................ 180

CVI seq 1 ............................................................ 180

CVI seq 2 ............................................................ 180

CVI seq 3 ............................................................ 180

Consensus EELCAQLCSTPPPPISTPHIIYAPGPSPLQPPICTPPPPDAEELCAQLCSTPPPPICTPH 240

CVI ............................................................ 240

CVI seq 1 ............................................................ 240

CVI seq 2 ............................................................ 240

CVI seq 3 ............................................................ 240

Consensus SLFCPPQPPSPEGIFPALCPVTEPCTPPSPGTVYAQLCPVGQAPLFTPSPPHPAPEPERL 300

CVI ............................................................ 300

CVI seq 1 ............................................................ 300

CVI seq 2 ............................................................ 300

CVI seq 3 ............................................................ 300

Consensus YARLTEDPEQDSLYSGQIYIQFPSDIQSTVWWFPGDGRP 339

CVI ....................................... 339

CVI seq 1 ....................................... 339

CVI seq 2 ....................................... 339

CVI seq 3 ....................................... 339

**JM102 Meq (vMeq)**

Consensus MSQEPEPGAMPYSPADDPSPLDLSLGSTSRRKKRKSHDIPNSPSKHPFPDGLSEEEKQKL 60

JM102 ............................................................ 60

JM102 seq 1 ............................................................ 60

JM102 seq 2 ............................................................ 60

JM102 seq 3 ............................................................ 60

Consensus ERRRKRNRDASRRRRRAQTDYVDKLHEAREELQRANEHLRKEIRDLRTECTSLRAQLACH 120

JM102 ............................................................ 120

JM102 seq 1 ............................................................ 120

JM102 seq 2 ............................................................ 120

JM102 seq 3 ............................................................ 120

Consensus EPVCPMAVPLTVTLGLLTTPHDPVPEPPICTPPPPSPDEPNAPHCSGSQPPICTPPPPDT 180

JM102 ............................................................ 180

JM102 seq 1 ............................................................ 180

JM102 seq 2 ............................................................ 180

JM102 seq 3 ............................................................ 180

Consensus EELCAQLCSTPPPISTPHIIYAPGPSPLQPPICTPPPPDAEELCAQLCSTPPPPISTPHI 240

JM102 ............................................................ 240

JM102 seq 1 ............................................................ 240

JM102 seq 2 ............................................................ 240

JM102 seq 3 ............................................................ 240

Consensus FYAPGLCSTPPPPISTPHIIYAPGPSPLQPPICTPPPPDAEELCAQLCSTPPPPICTPHS 300

JM102 ............................................................ 300

JM102 seq 1 ............................................................ 300

JM102 seq 2 ............................................................ 300

JM102 seq 3 ............................................................ 300

Consensus LFCPPQPPSPEGIFPALCPVTEPCTPPSPGTVYAQLCPVGQAPLFTPSPPHPAPEPERLY 360

JM102 ............................................................ 360

JM102 seq 1 ............................................................ 360

JM102 seq 2 ............................................................ 360

JM102 seq 3 ............................................................ 360

Consensus ARLTEDPEQDSLYSGQIYIQFPSDTQSTVWWFPGDGRPGGFRKLLESTSRAAAGPSIFHP 420

JM102 ............................................................ 420

JM102 seq 1 ............................................................ 420

JM102 seq 2 ............................................................ 420

JM102 seq 3 ............................................................ 420

Consensus GGVPGKCTQFAL 432

JM102 ............ 432

JM102 seq 1 ............ 432

JM102 seq 2 ............ 432

JM102 seq 3 ............ 432

**RB-1B Meq (vvMeq)**

Consensus MSQEPEPGAMPYSPADDPSPLDLSLGSTSRRKKRKSHDIPNSPSKHPFPDGLSEEEKQKL 60

RB-1B ............................................................ 60

RB-1B seq 1 ............................................................ 60

RB-1B seq 2 ............................................................ 60

RB-1B seq 3 ............................................................ 60

Consensus ERRRKRNRDAARRRRRKQTDYVDKLHEACEELQRANEHLRKEIRDLRTECTSLRVQLACH 120

RB-1B ............................................................ 120

RB-1B seq 1 ............................................................ 120

RB-1B seq 2 ............................................................ 120

RB-1B seq 3 ............................................................ 120

Consensus EPVCPMAVPLTVTLGLLTTPHDPVPEPPICTPPPPSPDEPNAPHCSGSQPPICTPPPPDT 180

RB-1B ............................................................ 180

RB-1B seq 1 ............................................................ 180

RB-1B seq 2 ............................................................ 180

RB-1B seq 3 ............................................................ 180

Consensus EELCAQLCSTPPPPISTPHIIYAPGPSPLQPPICTPPPPDAEELCAQLCSTPPPPICTPH 240

RB-1B ............................................................ 240

RB-1B seq 1 ............................................................ 240

RB-1B seq 2 ............................................................ 240

RB-1B seq 3 ............................................................ 240

Consensus SLFCPPQPPSPEGIFPALCPVTEPCTPPSPGTVYAQLCPVGQAPLFTPSPPHPAPEPERL 300

RB-1B ............................................................ 300

RB-1B seq 1 ............................................................ 300

RB-1B seq 2 ............................................................ 300

RB-1B seq 3 ............................................................ 300

Consensus YARLTEDPEQDSLYSGQIYIQFPSDTQSTVWWFPGDGRP 339

RB-1B ....................................... 339

RB-1B seq 1 ....................................... 339

RB-1B seq 2 ....................................... 339

RB-1B seq 3 ....................................... 339

**N strain Meq (vv+Meq)**

Consensus MSQEPEPGAMPYSPADDPSPLDLSLGSTSRRKKRKSHDIPNSPSKHPFPDGLSEEEKQKL 60

N strain ............................................................ 60

N strain seq 1 ............................................................ 60

N strain seq 2 ............................................................ 60

N strain seq 3 ............................................................ 60

Consensus ERRRKRNRDAARRRRREQTYYVDKLHEACEELQRANEHLRKEIRDLRTECTSLRAQLACH 120

N strain ............................................................ 120

N strain seq 1 ............................................................ 120

N strain seq 2 ............................................................ 120

N strain seq 3 ............................................................ 120

Consensus EPVCPMAVPLTVTLGLLTTPHDPVPEPPICTPPPPSPDEPNAPHCSGSQPPICTPRPPDT 180

N strain ............................................................ 180

N strain seq 1 ............................................................ 180

N strain seq 2 ............................................................ 180

N strain seq 3 ............................................................ 180

Consensus EELCAQLCSTPPPPISTPHIIYAPGPSPLQPPICTPAPPDAEELCAQLCSTPPPPICTPH 240

N strain ............................................................ 240

N strain seq 1 ............................................................ 240

N strain seq 2 ............................................................ 240

N strain seq 3 ............................................................ 240

Consensus SLFCPPQPPSPEGIFPALCPVTEPCTPPSPGTVYAQLCPVGQAPLFTPSPPHPAPEPERL 300

N strain ............................................................ 300

N strain seq 1 ............................................................ 300

N strain seq 2 ............................................................ 300

N strain seq 3 ............................................................ 300

Consensus YARLTEDPEQDSLYSGQIYIQFPSDTQSTVWWFPGDGRP 339

N strain ....................................... 339

N strain seq 1 ....................................... 339

N strain seq 2 ....................................... 339

N strain seq 3 ....................................... 339
