# Supplementary material for: Distinct polymorphisms in a single herpesvirus gene are capable of enhancing virulence and mediating vaccinal resistance
Source: PLoS Pathog. 2020 Dec 11;16(12):e1009104. doi: 10.1371/journal.ppat.1009104 (PMC7758048; doi:10.1371/journal.ppat.1009104)
Supplement: Attachment — Submitted filename: Meq rebuttal letter final.docx [file ppat.1009104.s006.docx]

**Response to referees**

We thank the reviewers for the careful evaluation of our work and provide a point-by-point response to the questions and comments.

Reviewer 1

**Part I - Summary**

This is a very straightforward paper where the authors investigated the long-believed association of Meq with MDV virulence. Using Red-mediated recombination and the infectious RB1B BAC clone, they inserted Meq genes from MD vaccine (Rispens) and three different MDV strains with defined virulence (v – JM/102W; vv – RB1B; vv+ - MK). These recombinant viruses were tested for pathogenicity and the ability to overcome HVT vaccinal protection. The results were the most surprisingly clean (especially Fig. 2B) that I’ve ever seen for MDV when birds are involved. And to my knowledge, this is the first MDV paper that associates more than one variant to disease incidence.

The authors took one next logical step, which was to determine what mechanisms might be involved in increased virulence. Using T cells that were stimulated by various agents, they convincing demonstrate that more virulent Meq isoforms can overcome the innate response that induce pathways involving TLRs or STING.

The main limitation is my opinion is that given the status of this journal, I would have liked to seen more experiments. Easy ones that come to mind include:

Biological replicates. This is traditional for MDV experiments given the variation that is normally observed by everyone else. I didn’t put this down as a Major Revision but…

Response 1: Thanks for this valuable comment. As suggested by the reviewer, we included data from an independent animal experiment performed by Dr. Parcells using a different chicken line. The data are comparable with the initially presented data and were included in the supplementary data (Fig. S1).

Competition assays, i.e., infect birds with two or more rMDVs to prove that the more virulent one does dominate, shed earlier and faster, etc. In my opinion, this is more definitive proof.

Response 2: This would indeed be interesting an animal experiment. However, as the recombinant viruses used in this study only differ by one to four amino acids, it would be very difficult to assess which virus dominates, especially as previous studies indicated that the competition assays commonly result in a mixed virus population. Intriguingly, Dunn et al. recently performed competition assays using strains from different pathotypes and evaluated virus competition [1]. They used either pathogenically similar or dissimilar virus pairs and could demonstrate that higher virulent strains had a competitive advantage over the less virulent strains. However, dominance of one strain over the other was not absolute for either virus pair and the subordinate virus was rarely eliminated. These experiments would be very exciting but are beyond the scope of this manuscript. We would be happy to collaborate on these experiments in case anybody is interested. To address this point, we discussed this aspect and the manuscript mentioned above in our discussion (lines 371-375).

Looking at the relative expression of Meq and especially the Meq/vIL8 splice variant. So do the variants influence how much and what isoforms are expressed?

Response 3: Great question. As suggested by the reviewer, we quantified the expression levels the *meq* isoforms. We did not observe differences in *meq* expression as shown in Fig. 1E-G. Moreover, we discussed the potential impact of splice variants in lines 290-294.

Analyzing which T cell population is being infected by each rMDV and addressing whether there are differences.

Response 4: The question whether different MDV strains (or different host genetics) have an effect on the predominantly infected T cell population is indeed a very intriguing one. However, this is beyond of the scope of this manuscript.

Finally the Discussion. This section just summarized the Results and not much else. So the lack of depth, speculation on how the Meq isoforms mechanistically affect virulence, etc. was disappointing especially considering the groups involved. Also as I recall seeing, the Parcells group has made a number of rMDVs with other forms of Meq that did not behave as expected. So while we always want to portray a positive picture, biology is never simple, so the authors should give a more balance discussion that includes potentially conflict results, the role of Meq/vIL8, acknowledge that there are probably other genes involved in the complex trait of virulence, etc.

Response 5: Thanks for pointing this out. We agree with the reviewer and extensively edited the discussion of our manuscript.

**Part II – Major Issues: Key Experiments Required for Acceptance**

As I wrote above, I was a bit disappointed in the Discussion. Not really sure if this qualifies but as a Major Issue but since it does not involve more experimentation, I think this limitation needs to be elevated especially since the status of the journal.

Response 6: Thanks for this valuable suggestion. As mentioned above, we worked on the discussion and implemented the reviewer’s suggestions.

**Part III – Minor Issues: Editorial and Data Presentation Modifications**

In no special order, these are the items that I believe would make this a better paper

Biological replicates. I waffled as to whether this should be a major issue especially since I’ve never seen such clean results with MDV in my life, especially those involving birds.

Response 7: As discussed above, we included replicate data from an independent animal experiments performed Dr. Mark Parcells (Fig. S1). The phenotype in the experiment was comparable to the ones shown in our initial submission. We also would like to point out, that all our animal experiments are performed in a blinded manner to eliminate the bias of the experimenters.

Title: Might qualify this a bit something like “Few polymorphisms in a single herpesvirus gene are capable of enhancing virulence and mediate vaccinal resistance.” I say this because I believe that virulence is a complex trait and there are likely many other existing variants beside those in Meq that contribute.

Response 8: Thanks for this comment, we changed the title to ”Distinct polymorphisms in a single herpesvirus gene are capable of enhancing virulence and mediate vaccinal resistance”.

Multiple places you say “vaccine protection.” I think “vaccinal protection” is more appropriate.

Response 9: Thanks, we have changed ‘vaccine protection’ to ‘vaccinal protection’ throughout the manuscript.

Lines 36-37. Possibly rephrase as “One virus that should shown repeated shifts to higher virulence in response to more efficacious vaccines has been the ...”

Response 10: Thanks for the suggestion, we have modified this sentence in the author summary as suggested.

Line 40. Replace “dictate” with “can significantly alter”

Response 11: We changed it accordingly.

Line 43. Delete “a” at the end of the line.

Response 12: Corrected

Line 48. You say some viruses but isn’t smallpox the only example?

Response 13: Smallpox and Rinderpest have been the only viruses eradicated. We changed the sentence to: ‘While vaccines are ideal interventions for eradication, some viruses can evolve to overcome vaccinal protection’.

Lines 52-29. This doesn't flow well. You just introduced that MDV is a well-known example of viral evolution to greater virulence. But then you give rambling facts. Why don't you first define what is MDV, what is the pathology, and how it has increased in virulence over time?

Response 14: Thanks for the suggestion. We improved the MDV introduction as suggested.

Line 63. While this may be true, it has never been really proven. What is true is that vvMDVs appeared ~10 years after the introduction of bivalent MD vaccine. I know that this is picky but "led" implies causative to me. There may have been other factors for increased virulence.

Response 15: We have removed the ‘led’ and restructured this paragraph along with the introduction of the different vaccines.

Line 73, Figure 1A. You give vaccine names but earlier you call them first, second, and third generation vaccines. Try to be consistent.

Response 16: We changed the vaccine names in the figure to first, second, and third generation vaccines to be more consistent.

Line 74. Replace “remains elusive” with “has never been proven”

Response 17: We replaced this phrase.

Line 76. Add a comma after “cells” to help separate the phrases

Response 18: We added the comma.

Line 88. Replace “instead of” with “to replace”

Response 19: Thanks, that sounds better.

Entire Results section. Strictly speaking, no introductory material, citations, interpretation, etc should be given in this section.

Response 20: We went through the Results section with this comment in mind. While we mostly agree, we believe that the remaining references in our Results section are indispensable. Moreover, we believe that the results paragraphs need a short overall description of the experiments.

Lines 102-111. This should be moved to the Methods section. Also it would be nice to separate the reads, coverage, etc. by Sanger from Illumina.

Response 21: Thanks for picking this up, we moved it to the methods section and noted that it was duplicated.

Line 117. I think you mean Fig. 1C

Response 22: Thanks, we corrected this error.

Line 119. Similarly, I think you mean Fig. 1D

Response 23: Corrected.

Line 135 and elsewhere. You use what I considered fairly high amounts of viral and vaccine pfu. Why? Is this because the chicken lines you use are genetically resistant? Remember that in pathotyping of MDV strains, ADOL and the vaccine industry use highly susceptible birds.

Response 24: As the reviewer anticipated, the VALO spf birds are relatively resistant to MDV. VALO chickens are closely related to a layer line that is used worldwide and therefore represent a good model for infections in the field. The infectious dose used in our study were optimized in several MDV animal experiments over the last decade.

Line 137. It might have been good to demonstrate that your qPCR assay worked equally well for all recombinant viruses since it appears you're using the same Meq probe.

Response 25: Good point. We used a primer and probe set that binds to a conserved region in *meq*. Nonetheless, to show that all the *meqs* have a comparable expression level, we performed qPCR as discussed above (Fig. 1E-G) and adjusted the methods and results sections accordingly.

Line 138. Bursal and thymic atrophy are more observed in more virulent MDV strains. Did you see this and if yes, then you should add this comment.

Response 26: Thanks for pointing this out. We did not observe obvious differences in the size on the bursa and thymus during our necropsies. However, bursal and thymic atrophy mostly occurs early upon infection (7-14 days), while the animals commonly succumbed to disease much later than that.

153. Are these tumors clonal? Not sure if this matters much but I would think higher virulent strains would lead to more non-clonal tumors.

Response 27: Interesting point, thanks! We performed next-generation sequencing analysis and only observed clonal tumors. There were no sub-populations observed.

Line 172 and Fig. 3B. This is a bit surprising as the vMeq should have caused disease. Also both the vvMeq and vv+Meq gave low disease incidence. Again, could this be due to using MD resistant birds?

Response 28: Yes, this is mostly due to using MD resistant birds. In addition, Fig. 3B shows data from naïve contact animals that are cohoused with the experimentally infected chickens since day one post hatching. These contact animals are naturally infected around day 14 of age and are thereby more resistant to disease than one-day old chickens.

Line 199 and Fig. 4D. A bit odd as HVT should protect only against v MDV strains; remember vv MDV strains are defined as the ability to evade HVT protection.

Response 29: It is true that HVT usually does not protect against vvMDV in susceptible chickens. In our MD resistant HVT-vaccinated chickens, we only observed clinical signs of MD upon infection with vvMDV (Figure 4C), but no tumors (Figure 4D). In addition, these birds were challenged day seven post-vaccination when the birds were older and more resistant to MDV. We discussed the important point in the discussion section (lines 328-331).

Line 224. You could made a stronger and more definitive statement about whether more virulent strains do transmit better is if you had performed mix infections.

Response 30: Thanks for the comment. As mentioned above, we further discussed this point in the context of the study of Dunn and colleagues [1]. We will also consider this aspect in future studies.

Lines 253. Consider changing “losses” to “costs” as the condemnation rates are very low these days.

Response 31: We changed the sentence, although given the insidious nature of the immunosuppression caused by MDV, losses due to this are difficult to parse and are likely still significant. Condemnation for secondary infections remains a significant issue.

Line 255. Consider changing “Nevertheless, MDV strains increased in virulence and overcame vaccine…” to “MDV strains have repeatedly increased in virulence and overcome vaccinal…

Response 32: We changed the sentence.

Line 258. Delete “could”

Response 33: Deleted.

Line 268. In fact, I would say deletion of Meq enhanced lytic replication, likely due to the inability of this rMDV to undergo latency.

Response 34: Thanks for bringing up this interesting aspect. We recently demonstrated that deletion of meq in RB-1B does not affect virus replication *in vitro* [2]. We included this information in the manuscript.

Line 280. Change to “positions” (plural)

Response 35: Changed.

Line 288. The correct name is JM/102W

Response 36: Thanks for this observation. We changed the nomenclature in the manuscript and in Fig. 1B.

Line 300. Suggest replacing “dissemination” with “tumor formation”

Response 37: Done.

Line 318-321. The Read et al. paper is important but one has to realize that the main reason more virulent MDVs shed more in vaccinated birds was that the unvaccinated ones died quickly. So this is what normally happens in the real world. Plus MD vaccines definitely reduce shedding in living birds. So to say that MD vaccines enhance viral shedding is incorrect.

Response 38: This is a good point. We changed the sentence accordingly (lines 366-368).

Line 354. It is traditional to give passage levels for all the viruses.

Response 39: We included the passage levels of all the viruses used in in vitro and in vivo experiments in the methods section. We never exceeded more than four passages for the viruses used in our study.

Line 392. For the contact birds, were these the same age and introduced into the cages as the original challenge birds. Not clear to me.

Response 40: We clarified this in the manuscript. As discussed above, the experimentally infected and contact animals hatched on the same day and were housed together.

Reviewer 2

**Part I - Summary**

The authors swapped out the meq genes from various pathotypes of MDV-1 and placed them in the RB1B (vv) backbone. They tested their recombinants for replication in vitro and in vivo, pathogenicity, tumor dissemination, and shedding. The strength of the manuscript is that the science is sound. The significant finding is that recombinants containing higher virulent meqs overcome innate immunity in vaccinated animals and shed better than those with lower virulent meqs. The research is proper but the manuscript is not worthy of the study and has numerous shortcomings.

**Part II – Major Issues: Key Experiments Required for Acceptance**

The weakness is the discussion or lack thereof. The discussion is mostly a summary of the results. There is no whole-genome sequencing data in the manuscript, nor relevant accession #s. There is no MK (vv+) sequence data available, and frankly, I believe they used the vv+ strain know as TK.

Response 1: We appreciated the constructive criticism of the reviewer and addressed these points in greater detail below. Based on the comments of all three reviewers, we drastically improved the discussion section. The accession numbers were included and the MK/N strain nomenclature clarified as discussed in the following points.

If the authors are not going to examine revertants, then they must provide whole-genome sequencing data. A proper paper would contain whole-genome data for virus stocks before animal studies and whole-genome data of virus from primary infected and contact birds (without or limited propagation in tissue culture).

Response 2: As suggested by the reviewer, we uploaded the whole-genome data of all the BAC sequences and the *meq* genes in Table S1. In addition, we performed next-generation sequencing on the virus stocks used for the study and a number of viruses from organs and tumors from infected animals as requested. The new data are summarized in Fig. S2 and Table S2.

Here are some issues that will make the manuscript publishable.

Major.

First of all, the title is not very scientific. What are a few? Higher than two but less than a handful? How about 4 (amino acids A, K, Q, and V)?

Response 3: We changed the title also based on the suggestion of this and other reviewers.

Lines 61 through 67. This is not informative to non-Marek's disease scientists. Please define what first, second, and third generation vaccines are. Turkey herpesvirus, GaHV-2, CVI988 (Rispens), etc.

Response 4: Thank you for the comment. We define first, second and third generation vaccines as suggested by the reviewer (lines 64-73).

Line 83. There is no MDV-1 strain called MK in the cited paper. Do you mean TK? Please give a GenBank accession # for the MK strain.

Response 5: Thanks for bringing up this important point. The ‘MK’ *meq* is also known as the N strain, whereas MK is the abbreviation of the farmer's last name (McKelvy). The N strain nomenclature was adopted throughout the manuscript to clarify this point. We also added the GenBank Accession number for the N strain in Table. S1. As a point of reference, mutations in the N strain are identical to those seen in strain 686.

Lines 109-111. Where are the accession numbers? Where is the sequencing data? Did all the recombinants have the same sequences outside the Meq loci?

Response 6: As suggested by the reviewer, all virus sequences are now accessible online, and the accession numbers were included in the revised version of our manuscript. Moreover, we included the GenBank accession numbers for all the *meq* genes used in this study (Table S1). The genomes of all recombinants were identical except for the mutations in the *meq* gene (Figure S2A and B).

Lines 143-145. vv meq is more virulent than vv+ meq. Why? This was not mentioned in the discussion. In essence, RB1B containing its ‘normal’ meq is more virulent than RB1B containing a vv+ meq. Isn’t that worth mentioning in the discussion?

Response 7: Thanks for bringing up this point. As suggested by the reviewer we discussed possible reasons in the discussion section (lines 317-321).

Lines 162-164. Four amino acids AKQV involved in a >50% increase in tumors. Shouldn’t this be mentioned?

Response 8: Yes, absolutely. We now further highlight findings in the discussion section (lines 279-281).

Lines 172-174. The vmeq RB1B recombinant in primary animals had ~ 10% tumors, but no tumors in vmeq RB1B contact birds? Did, or can the author sequence the MDV genome from the vmeq RB1B contact birds to determine what happened, especially to the meq gene? This was also not mentioned in the discussion.

Response 9: Great question. We sequenced the *meq* genes and performed next-generation sequencing on organ and tumor material from the birds. We observed no or only minor changes and included the results in the supplementary information (Figure S2). Importantly, experimentally and contact birds were hatched on the same day and housed together for the duration of the experiment. Therefore, the contact animals were infected much later (~ day 14) when they were already more resistant to MDV, providing an explanation for the 10 % reduction in tumor incidence. We discussed these points in the manuscript.

Line 248-249. Why does LPS affect vac/v differently than vv/vv+ relative to the induction by poly IC and cGAMP? Isn’t that interesting and worthy of mentioning in the discussion.

Response 10: The reviewer has a very keen perception. We mentioned this aspect in the discussion section as suggested by the reviewer in lines 388-392.

The discussion is basically a reiteration of the results, only lines 265-272 mention outside information.

Response 11: We agree with the reviewer and therefore drastically improved the discussion section, based on the comments from this and other reviewers.

Line 268. Lupiani used md5, which is a vv. How does deleting a vv meq not affect its replication in vivo? Isn’t that surprising?

Response 12: All recombinant viruses used in this study did encode one of the *meq* isoforms, providing an explanation why we did not observe the defect described by Dr. Lupiani for a *meq* deletion virus.

Line 288-290. This is complicated. Is this expansion region found in other meqs of lower and higher virulence? Jm102 has a 59 amino acid expansion, and CVI988 has a 60 aa expansion. Need accession numbers for these sequences you used.

Response 13: The expansion is found in lower virulent *meq* isoforms as well as in the CVI vaccine. We included the GenBank accession numbers in Table S1 as suggested by the reviewer.

Line 313 -318. This is just as a reiteration of results.

Response 14: Thanks, we worked on the discussion and remove the reiteration of the results.

Line 321- 326. How does oncogenic potential affect shedding??? Please come up with a hypothesis or something in the discussion.

Response 15: This is an exciting aspect that we included in the discussion section as suggested. Transformed T cell can efficiently reactivate the virus [3]. Transformation increases the number of these cells that can travel to the skin, resulting in the production and shedding of the virus. We discussed this point in lines 364-366.

The Material and methods lack the passage history to generate the MD stocks. How many times were the reconstituted viruses passed in cell culture before use in animal experiments?

Response 16: Thanks for this observation. We included the passage levels of all the viruses used in the methods section. We never exceeded more than four passages for the viruses used in our study.

Line 360. Accession number is EF523390.1 is for an RB1B that doesn’t transmit horizontally. Did you use the repaired RB1B BAC? Where is the reference for this?

Response 17: Excellent observation. We used the repaired RB-1B BAC generated by Jarosinski et al. [4]. Now and previously we performed next generation sequencing of the RB-1B strain and now uploaded its whole genome sequence on NCBI as a reference. Accession number MT797629

Where are the results of full genome sequencing? You need this when you don't examine revertants. What about secondary mutations.

Response 18: We included the next-generation sequencing results on GenBank (Table S1) as requested by the reviewer. In addition to the BACs, we also performed full genome sequencing on the recombinant viruses stocks used for the experiments and did not observe any mutations. The results from the data obtained are summarized in Fig. S2B.

**Part III – Minor Issues: Editorial and Data Presentation Modifications**

Line 68. Use ‘leaky’ This sounds like slang.

Response 19: We initially thought so too, but Dr. Read commonly uses this term in the context of MDV vaccines [5]. We further clarified the term in the manuscript in lines 75-76.

Lines 70-71. There should be references for this.

Response 20: Thanks, we added the missing reference.

Lines 106-108. This is confusing. You replaced the meq of vv RB1B with a different vv meq. It sounds like you are replacing RB1B meq with RB1B meq.

Response 21: We used a previously published delta *meq* RB1B virus (Conradie et al., 2019) and inserted the different *meqs* including the RB1B *meq*. We revised the sentence to clarify this point.

Lines 116-129. This is not labeled correctly. 1D is plaque size.

Response 22: Thanks. We corrected the label.

Line 181. There is no key for 3a. The x-axis is not continuous time. Some indication of the two groups is needed. Or make two figures.

Response 23: We added the key and split the figure (see Fig. 3A) as suggested by the reviewer.

Line 183. Red and orange problem. Please use more contrasting colors like red and blue.

Response 24: Yes, good point. We change the colors accordingly.

Line 191. Mention how the chickens were challenged. SQ?

Response 25: Yes, we challenged the chickens SQ and included this information in the text (line 149).

Lines 203-206. It is hard to read these graphs esp 4C. Difference between red and orange. Use purple or light blue?

Response 26: We changed the colors accordingly.

Line 354. This is not the correct reference on how to rescue an MDV BAC.

Response 27: Thanks for picking up on this. We corrected the citation.

Reviewer 3

**Part I - Summary**

In the manuscript by Conradie et al., the authors explore the idea that a single gene (the meq gene) may be largely responsible for the evolution of virulence (and the evolution of vaccine escape) that was observed over the past 50 years in a chicken pathogen, Marek’s disease virus (MDV). To explore this question, they make four recombinant viral strains that differ only in their meq gene, with each receiving either a vaccine type “vac”, a low virulence type “v”, a high virulence type “vv”, or a hyper virulence type “vv+”. They then perform in vitro and in vivo studies to show that the strains have different phenotypes, largely corresponding to what would be expected based on their type alone. Given that the meq gene is the only difference between these strains, the authors conclude that the meq gene is largely responsible for virulence and vaccine escape in the MDV system. This manuscript represents a large amount of work. The experiments are quite elegant, and the results are interesting. That said, I have two substantial issues that I think the authors need to directly address, before this would be appropriate for publication. In light of these two issues, I feel that the authors should probably also weaken some of their strongest statements (for example, lines 96-99 and 258-260).

Response 1: Thank you for the careful evaluation of our work. We thoroughly addressed the reviewer’s comments below and weakened the statements in the indicated lines.

**Part II – Major Issues: Key Experiments Required for Acceptance**

The first issue is that the authors use a single meq genotype taken from a single MDV isolate to represent the entirety of a pathotype (i.e. vac, v, vv, vv+). While this may be a reasonable assumption for the vaccine type, the other three pathotypes have population-level genetic variation within a single pathotype. Perhaps the authors have very good justification for selecting the strains that they have, but regardless, I am left wondering whether the same pattern would have been observed had they chosen, for example, a different representative “v” isolate. Obviously, it is unreasonable to expect the authors to repeat this analysis with a panel of genotypes, but I do think that this limitation of their study should be discussed.

Response 2: Thanks for giving us the opportunity to discuss this aspect. We selected these v, vv and vv+ genes as they are found in several strains of the respective pathotype. We therefore believe that our data is representative for a broach range of viruses and pathotypes, even though we cannot confirm this for every virus strain and *meq* sequence. In addition, we recently tested another mutant that harbors an alternative v *meq* isolate (617A) in collaboration with Dr. Parcells and obtained comparable results (Fig. S1).

The second issue is that all of these experiments were conducted in a single genetic background, the RB1B background, and so epistatic effects between meq and the rest of the genome were not controlled for. RB1B is a “vv” virus, and it also happens to be the same virus from which their “vv” meq was chosen. I am thus left wondering how much of their results are due to epistatic effects, where the fitness of the virus is impacted not by meq alone, but by its interaction with the rest of the viral genome. Sequence data show that vaccine isolates and low virulence v isolates tend to cluster together on one branch of a phylogenetic tree while higher virulence vv and vv+ isolates tend to cluster together on another branch. I therefore worry that some of their main conclusions may be the result of a “v” or “vac” meq simply not performing all necessary functions to maintain viral fitness in a “vv” background. Evidence to suggest epistatic effects can be seen in Figure 2A, 2B, and 2C, where the “vv” strain appears to be more virulent than the “vv+” strain, which could be explained by epistatic effects but not by the hypothesis posed by the author that the meq gene controls virulence. The observed low virulence of “v” and “vac” meq strains would be predicted by both the epistatic effect hypothesis and the hypothesis posed by the authors have the same prediction, and so it is difficult to conclusively determine which is responsible for their results. Again, I think it is unreasonable to expect the authors to repeat their set of experiments using a “v” or “vac” background, but I again think that it is necessary to discuss the limitations that result from experimental constraints.

Response 3: The reviewer raises a very interesting point. As suggested, we discussed the potential epistatic effects and the limitations of using one genetic background. These changes improved our discussion section and made our manuscript stronger.

**Part III – Minor Issues: Editorial and Data Presentation Modifications**

Introduction: I think the word “chickens” doesn’t appear until line 91, which is way after the system is introduced. I have no problem with trying to keep things general, but I do think it is necessary to say that MDV is a pathogen of chickens. For example, it seems like it would fit really well in line 52. “…Marek’s disease virus (MDV), a pathogen of chickens.”

Response 4: Thanks for the recommendation. We have introduced chickens as suggested by the reviewer.

Lines 109-111: Does this analysis ensure that the meq gene was inserted in the correct spot (two spots) and nowhere else? I am not familiar enough with these methods to know, but if so, the authors should say so.

Response 5: Thanks for bringing this up. We performed whole genome sequencing (Illumina MiSeq) to confirm that every nucleotide in the virus genome is correct (NGS of BAC and virus stocks) and that the *meq* gene is exactly in the right position. We clarified this aspect in the text (line 118-120) and included a figure, Fig. S2A and B.

Figure 1A (left side): My brain had trouble reading “low virulence pathotypes” at the top of the figure and “high virulence pathotypes” at the bottom of the figure, especially because that flips on the right side of this figure. Might be worth flipping.

Response 6: Great idea. We flipped it as suggested.

Figure 1A (right side): To my knowledge, vv++ strains have not yet been described, and so the arrow that extends beyond the CVI line is misleading. Perhaps a question mark could be added.

Response 7: Thanks for this suggestion. We included the question mark as suggested.

In many places throughout the text (i.e. 140-141, 150-151, 172-174, 277-278, there may be others that I missed), the authors state that the v meq or the vac meq “abrogated” or removed virulence or reduced oncogenicity. However, this wording is misleading, because although RB1B has high virulence, the Delta meq RB1B isolate has low virulence and oncogenicity. Adding the v meq therefore increased virulence and oncogenicity, just not by as much as adding a vv meq.

Response 8: The reviewer raises an interesting point. While a comparison to the delta meq RB1B is a good option, our next generation sequencing data allows us to compare the different viruses directly to wild type, as only a few amino acid changes in the meq gene are present in the entire virus genome. We carefully went over the manuscript to confirm our wording is correct for the respective comparison.

On lines 145-148, the authors state that the vv+ meq causes neurovirulence. This would be interesting given that vv+ isolates of MDV are typically associated with neurovirulence (suggesting that meq plays a role in neurovirulence) but the data are not presented. The data should either be presented, or the statement should be removed.

Response 9: We removed the statement.

Line 176: It should be stated that the “slightly enhanced” effect is not significant.

Response 10: We now stated this.

Figure 3 legend (lines 183-184): I found the wording here to be confusing. Are panels C and D showing data for experimentally-infected birds or for naturally-infected birds. If the latter, why is tumor incidence higher than disease incidence? That brings me to ask how you are defining disease incidence in general (is it mortality and/or reaching clinical endpoints?)? If so, that should be stated.

Response 11: Thanks. We clarified this in the manuscript. Figure 3 shows the data of naturally infected contact animals. Disease incidence is defined by reaching a set clinical endpoint (e.g. somnolence, severe ataxia, death) during the course of the experiments prior to termination. Tumor incidence also includes the animals that showed clear gross tumors during final necropsy, and can, therefore, be higher than the disease incidence. We clarified this in the text.

Figure 3: I was also wondering whether disease incidence is significantly different between pathotypes. I can see that the pattern is in the direction that would be expected based on pathotype, but I cannot determine from the figure whether the vv and vv+ isolates are significantly more likely to cause disease than v or vac isolates.

Response 12: Good point. The disease incidence of vv+Meq (Fig. 3B) is significantly different compared to v and vac. The asterisks were included in the figure.

Line 189 (or possibly in methods): Are these chickens maternal antibody positive or maternal antibody negative.

Response 13: All chickens were maternal antibody negative.

Figure 4 legend (line 211): Should “vacMeq” be “vvMeq”? Based on the figure it appears that it should. If not, the tables below Fig 4E and 4F are either wrong or I am misinterpreting how to read them.

Response 14: Thanks for picking this typo. We corrected it in the manuscript.

Figure 5B: Relative plaque sizes differ between the different virus strains, and so correcting for plaque size is a little misleading. The plaques may actually be very similar sizes between vacMeq and vv+Meq in the immune activated treatments, but because of differences in the reference (non stimulated) treatment, it would look like the different meq variants are altering ability to escape innate immunity. I would want to see the raw data based on actual plaque size rather than corrected plaque size.

Response 15: We changed the figure to indicate the original plaque sizes of all the viruses, normalized to vvMeq instead, and we also included the average of the actual plaque size of three independent experiment here:

Line 258: Delete “could”.

Response 16: We deleted the word.

Lines 262-264: This sentence needs to be reworded to be grammatically correct.

Response 17: We corrected the sentence.

Lines 281-282: The Rispens vaccine has a large insert. Was this insert included in the “vacMeq”? If so, the sentence on lines 281-282 is misleading. If not, why was the full Rispens meq gene not used? Regardless, you should be clear about the sequence that was inserted. Perhaps full meq genes could be included as supplemental info.

Response 18: Thanks for raising this point. The Rispens vaccine has two predominant *meq* isoforms expressed, the long- and short *meq* isoform. In this manuscript, we characterize the short form, as we had already previously investigated the long form (Conradie et al., 2019). We clarified this in the text and included the GenBank accession numbers in the supplementary of all the *meq* genes used in this study - Table S1.

Lines 288-289: Again I feel that this sentence may be misleading unless I am confused. My understanding is that JM102 does not have a 21 amino acid insertion, but rather a series of point mutations that make it differ from typical vv and vv+ isolates. Providing the full sequences for the meq regions inserted is needed.

Response 19: We clarified the sentence and included the GenBank accession numbers for all *meq* genes in Tab. S1.

Lines 294-295: Was this difference statistically significant?

Response 20: Yes, they are now indicated by asterisks.

Lines 389-391: There are missing details here. How were animals housed? As a reader, I am curious about their space, airflow, disinfection between experiments, etc. Also, did any animals need to be culled for space reasons, or did any animals die from anything other than MD? If so, how did you deal with this in your statistical analyses?

Response 21: We included the information requested by the reviewer in the methods section. Each group of animals were housed in isolation units with incoming and outgoing air filtered through HEPA filters. Units are cleaned with a validated protocol using disinfectants and subsequent H_2_O_2_ sterilization. The rooms are exclusively used for our MDV infection experiments and incoming material is sterilized. Each room has two stainless steel cages with a total of four square meters of space and a total capacity of 36 adult chickens (>1,2 kg). Therefore, no animals had to be culled for space reasons. Each group started with 25 experimentally infected and 11 contact chickens. A maximum 1 or 2 animals per group died of early mortality (until day 4) or due to picking, resulting in an n of 23 -25 each group (see manuscript). These animals were excluded from the study as they did not show clinical signs of MD or other diseases.

References:

1.	Dunn JR, Silva RF, Lee LF, Witter RL. Competition between two virulent Marek's disease virus strains in vivo. Avian Pathol. 2012;41(3):267-75. doi: 10.1080/03079457.2012.677804.

2.	Bertzbach LD, van Haarlem DA, Hartle S, Kaufer BB, Jansen CA. Marek's Disease Virus Infection of Natural Killer Cells. Microorganisms. 2019;7(12). Epub 2019/11/24. doi: 10.3390/microorganisms7120588. PubMed PMID: 31757008.

3.	Kaufer BB, Jarosinski KW, Osterrieder N. Herpesvirus telomeric repeats facilitate genomic integration into host telomeres and mobilization of viral DNA during reactivation. J Exp Med. 2011;208(3):605-15. doi: 10.1084/jem.20101402. PubMed PMID: 21383055; PubMed Central PMCID: PMCPMC3058580.

4.	Jarosinski KW, Margulis NG, Kamil JP, Spatz SJ, Nair VK, Osterrieder N. Horizontal transmission of Marek's disease virus requires US2, the UL13 protein kinase, and gC. J Virol. 2007;81(19):10575-87.

5.	Read AF, Baigent SJ, Powers C, Kgosana LB, Blackwell L, Smith LP, et al. Imperfect vaccination can enhance the transmission of highly virulent pathogens. PLoS Biol. 2015;13. doi: 10.1371/journal.pbio.1002198.
