# Supplementary material for: Distinct polymorphisms in a single herpesvirus gene are capable of enhancing virulence and mediating vaccinal resistance
Source: PLoS Pathog. 2020 Dec 11;16(12):e1009104. doi: 10.1371/journal.ppat.1009104 (PMC7758048; doi:10.1371/journal.ppat.1009104)
Supplement: Attachment — Submitted filename: Rebuttal Final 2.docx [file ppat.1009104.s007.docx]

**Response to referees**

We thank the reviewers for another careful evaluation of our work and provide a point-by-point response to the questions and comments.

Reviewer 1

**Part I – Summary**

I like the revisions that the authors provided especially the inclusion of NGS to verify that there were no unexpected changes in the viral genome. I also agree with the major conclusions, which are (1) small amino acid changes can have large effects on virulence. (2) Meq isoforms are associated with evading the innate immune response.

**Part II – Major Issues: Key Experiments Required for Acceptance**

A more balanced discussion that indicates among others (1) virulence is a complex trait, (2) Meq isoforms do not correlate with virulence of the origin strain, and (3) a comparison of Meq splice variants, especially with vIL8), among the isoforms.

Response 1: We addresses these points as follows:

Point (1): Thanks for raising this point. As suggested, we now discuss that virulence is a complex trait and that a number of virulence factors are involved in MDV pathogenesis in the manuscript (line 225-229).

Point (2): This is a valuable suggestion and we discussed the limitation of our study in the manuscript (line 276-288)

Point (3): Good point. We clarified in the manuscript that the mutations in the *meq* isoforms do not alter the splice sites (donor, acceptor and branch point). To validate this on an experimental level, we performed additional experiments and demonstrate that splicing with vIL8 is not affected (Figure 1, line 128-133 and 240-250).

**Part III – Minor Issues: Editorial and Data Presentation Modifications**

Title. Should it be “mediating” and not “mediate” as this follows after “capable of”?

Response 2: Thanks, we changed this to mediating. Line 2

Lines 74 and 75. It is “MD,” not “MDV” vaccine. Vaccine is to the disease, not the virus.

Response 3: Thanks for picking up on this and we changed it accordingly. Line 73

Lines 83 and 86. Why do you write “b-ZIP” vs. “bZIP” (no hyphen), which is what I think is more traditional?

Response 4: We removed the hyphen as suggested. Line 82 and line 85

Lines 129-131 and Figure 1 (E-G). I’m trying to understand this. Is Figure 1E and !F, the amount of ICP4 and GADPH copies? If so, then how did you determine this? And Figure 1G is relative Meq levels but how did you normalize to both ICP4 and GAPDH (lines 461-463)? I’m confused as when I do relative expression, I do delta delta delta Ct analyses using one normalizing gene. Also, to determine the relative expression of a gene, the normalizing gene expression should be relatively static AND close to the level of expression of the test gene to provide sufficient accuracy. I know that this is hard, if not impossible, with an infected cell but if the relative levels are 10e5 or above, then I doubt you have much power in comparing between samples.

Response: 5 Thanks for this very keen observation. We used standard curves for an absolute quantification of the genes by PCR and presented the data as *meq* relative to GAPDH. The ICP4 copies were displayed to validate that all cultures were infected with the same pfu of the respective viruses. We moved the ICP4 and GAPDH control graphs to the supplementary data (Fig. S3) to avoid confusion. We changed the manuscript, the materials and methods section and the legend of Figure 1 accordingly (line 435-437 and line 841-845).

Also, given that you performed resequencing of the viral genomes, I would think looking at differences in Meq splice variants, especially the one with vIL8, would not be that difficult.

Response 6: Thanks for this comment. As suggested, we performed RT-PCR which revealed that *meq*/vIL8 splicing is not affected. We included the new date in Figure 1 and discussed the *meq*/vIL8 splice variants in line 128-133 and 240-250.

Lines 164-165. Figure 2D shows that vvMeq has a significantly higher tumor incidence compared to vv+Meq, and no difference in tumor number. Please revise.

Response 7: We revised the manuscript as suggested by the reviewer (line 149-152 and line 854-855).

Line 167. I think you mean different chicken “line” or “genetics.”

Response 8: Yes, we corrected it to “chicken line”. Line 156

Figures 3 and S1. How can the tumor incidence be higher than the disease incidence for vvMeq and vv+Meq as tumors are one of the main criteria for disease?

Response 9: Disease incidence is display as the number of animals that succumbed to disease within this 91-day animal experiment. The tumor incidence is displayed as all animals that developed tumors through the course of the experiment and the ones observed during final necropsy. This is now clarified in the manuscript (line 149-151)

Line 216, Figure 4B. It has been repeatedly reported that HVT does not replicate well in chickens, which is contrary to your results. So what controls did you perform to show that your HVT primers to SORF1 do not amplify MDV sequences? One simple test might be to screen contact birds that were co-housed with HVT-vaccinated birds. The thought is that since HVT replicates poorly, it is also known that it rarely transmits horizontally.

Response 10: As suggested by the reviewer, we validated our primer the primer/probe using samples from RB-1B and HVT infected animals from an ongoing animal experiment (see figure below). The HVT genome (green, n=8) was efficiently detected in chickens vaccinated with HVT, but the PCR was negative for animal infected with RB-1B alone (pink, n=8).

These qPCR analyses revealed that the primers are highly specific for HVT and do not detect RB-1B, which confirms the data of Dr. Nair’s group who developed this qPCR assay [1, 2]. These points are discussed in line 486-488

Line 282. While the 50+% increase was observed, I think you should provide some moderation by saying something like “in our experiments” as you only tested one background virus.

Response 11: We changed it to ‘In our experiment’ as recommended (line 234).

Lines 292-294. I agree that Meq variants did not affect splicing in B cells but what about CD4 T cells, which are the primary transformation target cell type? You’re pretty much discounting that Meq variants have no influence on splicing, which may out to be true, but not proven. Would like some wording to softening your dogmatic-like claim.

Response 12: To address the reviewer’s comments, we investigated splicing of meq/vIL8 in both CECs and CD4 T cells. We did not detect differences in the meq/vIL8 splicing, which is consistent with the absence of changes in the splice sites. We included the data (see Figure 1) and discuss it in the discussion section (line 242-250).

Line 319. You say that the disease incidence for vv+Meq was not significantly lower compared to vvMeq in Figure 2B. However, Figure S1A shows a highly significant difference.

Response 13: We removed this claim as requested by the reviewer.

Lines 365-366. Differences in feather load among various recombinant MDV strains appears as early as 10 days and certainly by 14 days. While transformation this early is possible, do you really think that this is the major driving force for the increased viral copies? This is highly speculative. And alternative theory is that different Meq isoforms replicate better in the feather follicles?

Response 14: Thank you for providing this alternative explanation which we included in the discussion section (line 332-336).

Discussion. Still no commentary about how different Meq isoforms lead to varying results. For sure, the authors have demonstrated that their Meq isoforms can lead to higher virulence though not exactly as predicted. This limitation as well as other work from the Parcells group needs to be incorporated. This is not intended to negate the work but rather that it is still difficult to predict the phenotypic outcome of recombinant MDVs even when incorporating variants of a major gene like Meq.

Response 15: Thank you for raising this point which we intensively discussed with Dr. Parcells. There is broad range of aspects that the mutations observed in *meq* as a transcription factor may affect including the induction or repression of different cellular proteins, the post-translational modifications on these proteins (ubiquitination, phosphorylation, etc.), and in turn appear to have very different effects on MDV pathogenicity. These variations differ between strains in which these changes evolved. We have observed this previously, when we inserted *meq* of RB-1B into rMd5, which altered the phenotype of that virus in the subtle way and allowed the establishment of tumors cell lines (UD36, UD37, UD38) which could not be achieved for rMd5 (Kumar, et al., 2012). The RB-1B Meq had three amino acid differences compared to rMd5. rMd5, although classified as a vvMDV, has a different pathogenesis in chickens as it is more inflammatory, causes greater immune organ damage, higher mortality and tumor incidence. Tumors induced by the recombinant showed similar cellular profiles to rMd5, suggesting to us at that time (which we mentioned in our results and discussion) that the context of the Meq protein (in terms of the strain in which it is encoded) plays an important role in tumor development, as well.

We also know this from our testing of a RB-1B Meq deletion (RB-1B∆Meq) mutant compared to rMd5∆Meq in terms of the replication of these viruses in vivo and their comparative ability to serve as vaccines. RB-1B∆Meq replicated to a much lower level during the early lytic phase and caused none of the thymic or bursal damage observed with rMd5∆Meq. rMd5∆Meq has been determined to be one of the best vaccines in the context of broiler chickens (with maternal antibodies). So, clearly the context of the Meq is important. We included a compact version of these pointe in the manuscript (line 276-288)

**Side comments:**

Competition assays do not rely on aa changes and/or antibodies only. One can easily compare the relative amount of viral genomes given known DNA polymorphisms that discriminate between strains.

Response 16: Thanks for pointing this out

My question on clonality cannot be addressed by looking at viral genomes. The best method, to my knowledge, is T cell receptor (TCR) spectratyping. Using this assay, you can determine if the tumor was VB1 or VB2, and if the dominant peak are different, then assume that the tumors are not clonal. The point is that more virulent strains might be more equipped to overcome the immune response, which would lead to more viral transformations early on.

Response 17: Thanks for this valuable suggestion. We would love to do TCR spectra typing in future studies and are happy to collaborate on this aspect.

If Meq isoforms do not significantly alter viral replication both in vitro and in vitro, yet alter disease and tumor incidence, what does this say about the major mode of activity? In my opinion, the observable effects are on the host genome.

Response 18: Yes, this is indeed an intriguing observation and futures studies should further address the effects on the host genome.

Reviewer 2

**Part I – Summary**

The is the second review of the manuscript and it is much improved especially the discussion section.

**Part II – Major Issues: Key Experiments Required for Acceptance**

The experiments are acceptable.

**Part III – Minor Issues: Editorial and Data Presentation Modifications**

Line 166. The data of this in vivo experiment was validated in an independent animal experiment using a different chicken. Add the word “line” after chicken.

Response 1: We added the word ‘line’. Line 156

Line 307 Meq binds to its own promoter, and by with weak transactivating properties, the vacMeq could in turn cause low levels of 308 Meq expression in vivo and the development of T cell tumors might fail to occur. Replace “by with” with the word “through”

Response 2: Thanks, we made the change as suggested. Line 264

Reviewer 3

The authors addressed my criticisms in the text of their response letter, but I do not see adequate changes to the text of the manuscript to address these concerns for future readers. In particular, my two major criticisms were that 1) they used only a single version of meq to represent each pathotype, and 2) they used only a single pathogen background and so cannot definitely rule out epistatic effects. With regard to 1, they wrote the following reasonable response but did not include it in the manuscript text. A version of this text needs to be included in the discussion:

"Thanks for giving us the opportunity to discuss this aspect. We selected these v, vv and vv+ genes as they are found in several strains of the respective pathotype. We therefore believe that our data is representative for a broach range of viruses and pathotypes, even though we cannot confirm this for every virus strain and meq sequence. In addition, we recently tested another mutant that harbors an alternative v meq isolate (617A) in collaboration with Dr. Parcells and obtained comparable results (Fig. S1)."

Put it into the manuscript if not already – add line numbers

Response 1: We apologize for this mix-up. As requested by the reviewer, we included version of this text in the manuscript (line 346-350).

With regard to point 2, the authors wrote that they responded, but I do not see a response. They wrote in the letter: "As suggested, we discussed the potential epistatic effects and the limitations of using one genetic background. These changes improved our discussion section and made our manuscript stronger." However, unless I am missing where they responsed (please include line numbers in response letters in the future), in the text, they do not adequately address this issue. In fact, the only time they use the term "epistatic" is in justifying why the vv+Meq strain does not behave as expected in one case. However, it is critical that they also explain that the possible existence of epistatic effects limits their conclusions.

Response 2: We apologize that this point was also missed potentially due to the extent of the previous rebuttal letter (24 page). This point was included in the manuscript as suggested by the reviewer (line 276-280).

Also, I asked the reviewers to qualify that one of their observations was nonsignificant. In the previous version that was line 176, and they say in their response letter "Response 10: We now stated this.". In the updated manuscript, this text now appears on lines 164-165. It reads, "In contrast, vv+Meq even showed an enhanced tumor dissemination pattern compared to the very efficient vvMeq (Fig. 2D)." This effect is non-significant. The authors cannot say this without stating that it is not a significant difference. It is very frustrating as a reviewer to have a comment ignored that is this easy to address.

Change sentence & provide line number

Response 3: Thanks for raising this point. We changed the sentence to comply with the reviewer’s request (line 153-154).

Line 29: "increase" needs to change to "modify" or "decrease" since the authors have only shown that moving from vv to v or vac meq decreases virulence, and not the other way around.

Response 4: Thanks for raising this point. We changed increase to ‘alter’. Line 28

Line 30: Delete "field" since this study wasn't done in the field or done using "field strains".

Response 5: True, we deleted ‘field’.

Line 101-102: This result should be discussed given that "vv" isolates are expected to be able to escape vaccine protection but you did not see that.

Response 6: We used VALO SPF birds in our study that are relatively resistant to MDV. In addition, these birds were challenged day seven post-vaccination when the birds were older and more resistant to MDV. In these resistant HVT-vaccinated chickens, we did observe clinical signs of MD upon infection with vvMDV (Figure 4C), but no tumors (Figure 4D). We further discussed this point in line 308-312.

Line 154 and 162: "Insertion" is misleading and should be changed. "Replacing the vv meq with v meq" may work better because meq isn't inserted, but rather replaced.

Response 7: Thanks for the recommendation. We changed ‘insertion’ to “replacement with the”. Line 151

Fig2D: Are you plotting the mean number of tumors or the mean number of tumors given the presence of at least one tumor? You say the former, but I suspect it is the latter and should be fixed.

Response 8: Thanks for pointing this out, and you are right. It is the latter and we clarified this in line 854-855.

Line 167: Add "breed" after "chicken".

Response 9: Thanks, we corrected this to “chicken line”, as requested by the other two reviewers. Line 156

Line 279: Delete "minor", since "minor polymorphism" has a very specific meaning and it is not the meaning meant here.

Response 10: Thanks for the comment. We changed minor to ‘distinct’ to resemble the title. Line 231

Line 307: I do not understand what "and by with weak transactivating properties" means. Please rewrite.

Response 11: We clarified this in the text now. It has been previously shown that the Meq from the CVI988/Rispens vaccine is a weaker transactivator corresponding to a decreased cellular and viral gene expression (line 261-264). An example of a viral gene transactivated is Meq itself, follows that is explained in line 264-267.

Line 309: "mutations" should be "mutation"

Response 12: Thanks for this grammatical point, we removed the ‘s’.

**Part II – Major Issues: Key Experiments Required for Acceptance**

No comments

**Part III – Minor Issues: Editorial and Data Presentation Modifications**

No comments

**References**

1.	Islam A, Cheetham BF, Mahony TJ, Young PL, Walkden-Brown SW. Absolute quantitation of Marek's disease virus and Herpesvirus of turkeys in chicken lymphocyte, feather tip and dust samples using real-time PCR. J Virol Methods. 2006;132(1-2):127-34. Epub 2005/11/18. doi: 10.1016/j.jviromet.2005.10.009. PubMed PMID: 16290211.

2.	Islam A, Harrison B, Cheetham BF, Mahony TJ, Young PL, Walkden-Brown SW. Differential amplification and quantitation of Marek's disease viruses using real-time polymerase chain reaction. J Virol Methods. 2004;119(2):103-13. doi: 10.1016/j.jviromet.2004.03.006. PubMed PMID: WOS:000221978500007.
